
JOURNAL INFORMATION
==============================
NLM Title Abbreviation: Biospektrum (Heidelb)
Journal ID: Biospektrum (Heidelb)
NLM Journal ID: 9615685

Biospektrum

ISSN: 0947-0867
EISSN: 1868-6249

ARTICLE INFORMATION
==============================
PMCID: PMC8501362
PMID: 34658540
DOI: 10.1007/s12268-021-1649-6
Article ID: 1649
Article version: 1
Subjects: Biotechnologie

Automation der Bestimmung von Vitamin D in Serum und Plasma

Thurow Kerstin kerstin.thurow@celisca.de http://www.celisca.de
1
Fleischer Heidi 2
Bach Anna 2
1 grid.10493.3f 0000000121858338 Center for Life Science Automation CELISCA, Universität Rostock, F.-Barnewitz-Straße 8, D-18119 Rostock, Deutschland
2 grid.10493.3f 0000000121858338 Institut für Automatisierungstechnik, Universität Rostock, Rostock, Deutschland
Electronic publication date: 2021 Oct 9
Print publication date: 2021
Volume: 27
Issue: 6
First page: 660
Last page: 662
Copyright: © Die Autorinnen 2021
License: Dieser Artikel wird unter der Creative Commons Namensnennung 4.0 International Lizenz veröffentlicht, welche die Nutzung, Vervielfältigung, Bearbeitung, Verbreitung und Wiedergabe in jeglichem Medium und Format erlaubt, sofern Sie den/die ursprünglichen Autor(en) und die Quelle ordnungsgemäß nennen, einen Link zur Creative Commons Lizenz beifügen und angeben, ob Änderungen vorgenommen wurden. Die in diesem Artikel enthaltenen Bilder und sonstiges Drittmaterial unterliegen ebenfalls der genannten Creative Commons Lizenz, sofern sich aus der Abbildungslegende nichts anderes ergibt. Sofern das betreffende Material nicht unter der genannten Creative Commons Lizenz steht und die betreffende Handlung nicht nach gesetzlichen Vorschriften erlaubt ist, ist für die oben aufgeführten Weiterverwendungen des Materials die Einwilligung des jeweiligen Rechteinhabers einzuholen. Weitere Details zur Lizenz entnehmen Sie bitte der Lizenzinformation auf http://creativecommons.org/licenses/by/4.0/deed.de.
License URL: https://creativecommons.org/licenses/by/4.0/

issue-copyright-statement: © The Author(s), under exclusive licence to Springer-Verlag GmbH Deutschland, ein Teil von Springer Nature 2021

==============================
Vitamins play an important role in many processes in the human organism. The detection of insufficient supply of vitamins is therefore of particular importance to avoid significant effects for human health. An increasing number of tests is only possible with suitable automated procedures. For the determination of vitamin D3 and vitamin D2 in serum samples, three methods were automated and compared with regard to their performance. All three methods enable reliable detection of 25(OH)D2 and 25(OH)D3 in serum in the ng/ml range.

■ Vitamine sollten ein essenzieller Bestandteil unserer Ernährung sein. Mangelversorgung mit Vitaminen kann zu unterschiedlichen Krankheitsbildern führen. Ist eine ausreichende Zufuhr über die Nahrung nicht möglich, muss u. U. eine zusätzliche Gabe von Vitaminen erfolgen. Vitamin D, das zu den fettlöslichen Vitaminen zählt, ist ein wichtiger Teil des Calciummetabolismus [1, 2]. Ein Mangel kann zu einer gestörten Knochenmineralisation (Rachitis bei Kindern, Osteomalazie bei Erwachsenen), Muskelkrämpfen, Migräne oder einer allgemein erhöhten Infektanfälligkeit führen. Diskutiert werden auch Zusammenhänge zwischen einem niedrigen Vitamin-D-Serumspiegel und SARS-CoV-2-Infektionen.

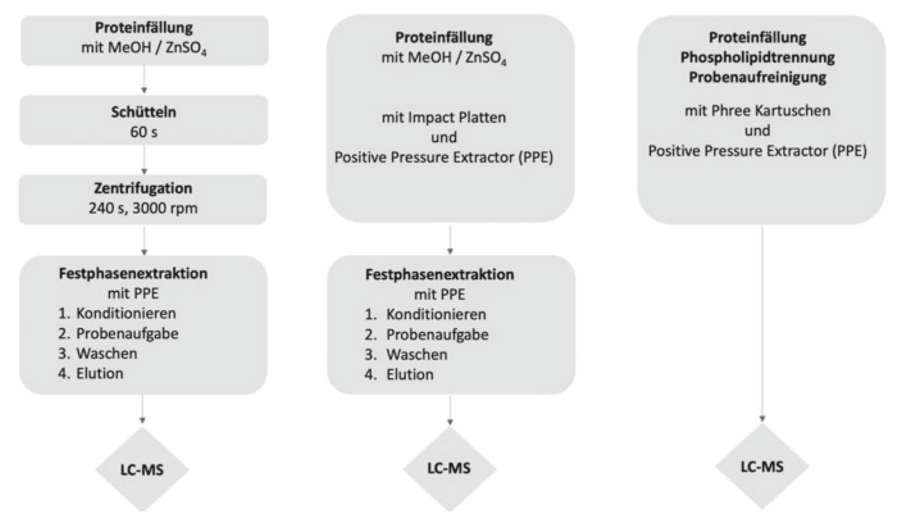

▲ **Abb. 1:** Vergleich der drei methodischen Ansätze zur Bestimmung von Vitamin D in Blut. Links: Methode 1 mit klassischer Zentrifugation; Mitte: Methode 2 mit Proteinfällung über Impact-Platten; rechts: Methode 3 mit Phree-Kartuschen (kombinierte Proteinfällung, Entfernung von Phospholipiden, Festphasenaufreinigung).

Vitamin D wird im menschlichen Organismus im Blut transportiert. Der Referenzwert für einen normalen Vitamin-D-Gehalt liegt zwischen 35 und 60 ng/ml Blut [3]. Der Umfang von Vitamin-D-Testungen hat sich aufgrund dessen Bedeutung in den vergangenen Jahren stark erhöht [4]. Für die Bestimmung von Vitamin D in Plasma oder Serum ist eine umfangreiche Probenvorbereitung erforderlich. Zunächst erfolgt eine Proteinfällung, die neben der Entfernung der Proteine für die nachfolgende analysenmesstechnische Bestimmung vor allem der Abspaltung des Vitamin D von seinem Trägerprotein dient. Hierzu werden üblicherweise Methanol, Acetonitril oder Mischungen [5], seltener auch Zusätze wie Zinksulfat eingesetzt [6]. Nach erfolgter Zentrifugation wird der Überstand zur weiteren Isolation und Aufkonzentration des Analyten einer Extraktion unterzogen; dies können sowohl Flüssig-Flüssig-Extraktionen [7] als auch Festphasenextraktionen sein [8]. Anschließend erfolgt i. d. R. ein Eindampfen zur Trockne sowie eine Wiederaufnahme in einem für die messtechnische Bestimmung geeigneten Lösungsmittel.

Für eine weitere Steigerung der Testzahlen ist die Automatisierung dieser arbeitsintensiven Prozesse eine wichtige Voraussetzung.

## Automatisierung der Bestimmung von Vitamin D

Für die Automatisierung der Bestimmung von Vitamin D wurde eine SPE-basierte Methode gewählt. Hierbei werden die Proben (200 µl Serum) nach Zugabe von 40 µl internem Standard ($D_6$-25-Hydroxyvitamin D3; 50 µg/ml), 500 µl Methanol und 200 µl 0,2 M Zinksulfatlösung geschüttelt und anschließend zentrifugiert. Ein Aliquot des Überstands (500 µl) wird einer Festphasenextraktion zugeführt; hierfür kommen Strata C8 1-ml-Kartuschen (Phenomenex, Torrance, US) zum Einsatz. Jeweils 10 µl der nach der Elution mit 2 × 100 µL Methanol resultierenden Lösungen werden direkt der Analytik mittels LC/TOF-MS zugeführt. Als Trennsäule wurde eine Zorbax Eclipse PAH (Rapid

Resolution HT, 4,6 × 50 mm, 1,8 µm) eingesetzt. In der ersten Automationslösung erfolgte eine 1:1-Umsetzung dieses manuellen Verfahrens. Hierzu wurde eine Zentrifuge in das Automationssystem integriert. In einem zweiten Ansatz wurde der Proteinfällungsschritt durch den Einsatz spezieller Platten für die Proteinfällung vereinfacht (Impact Protein Precipitation 2 ml 96 well filter plates, Phenomenex). Ein Aliquot der Probe (200 µl) und 40 µl des internen Standards werden dabei auf die Fällungsplatten aufgebracht, anschließend erfolgt die Zugabe von 500 µl Methanol und 200 µl 0,2 M Zinksulfatlösung als Fällungsreagenz. Die Abtrennung der Proteinmatrix erfolgt dann durch eine Filtration durch die Fällungsplatten. 500 µl des Filtrats werden wie im ersten Ansatz einer weiteren Aufreinigung über eine Festphasenextraktion zugeführt; die resultierenden Lösungen anschließend mittels LC/TOF-MS vermessen.

Im dritten Ansatz wurde eine weitere Vereinfachung gewählt, indem spezielle Platten eingesetzt wurden, die neben der Proteinfällung auch eine weitere Abtrennung von Phospholipiden ermöglichen (Phree Phospholipid Removal 1 ml tubes; Phenomenex). Dazu wurden 100 µl Serum, 50 µl interner Standard sowie 850 µl Acetonitril mit 0,1 % Ameisensäure auf die Kartuschen aufgegeben und durch mehrfaches Aspirieren und Dispensieren gemischt. Nach einer Inkubationszeit von drei Minuten wurden die Proben von 500 mbar filtriert. Eine weitere Aufreinigung der Proben durch eine nachfolgende SPE kann dann entfallen, wodurch das Verfahren schneller wird. **Abbildung 1** zeigt eine vergleichende Gegenüberstellung der schematischen Abläufe der drei Verfahren.

## Systemdesign

Die Automationslösung sollte möglichst platzsparend sein, damit sie in normalen Laborräumen ohne zusätzlichen Aufwand nutzbar ist. Als Basis wurde daher ein klassisches Liquidhandling-System (Biomek i7, Beckman Coulter Life Sciences) eingesetzt. Für den Zentrifugationsschritt wurde eine Mikrotiterplatten-Zentrifuge VSpin (Agilent Technologies) in das System integriert (**Abb. 2**, 1). Die Festphasenextraktion für die Aufreinigung der Proben erfolgt über einen integrierten automatisierten Positive Pressure Extractor (amplius GmbH) (2). Das System ermöglicht eine Druckbeaufschlagung von SPE-Kartuschen oder -platten mit bis zu 7 bar. Durch eine zusätzliche Flussreduzierung kann ein Austrocknen der Kartuschen vermieden werden. Der PPE wird durch den Gripper des i7 mit Labware bestückt, der Transfer der Proben erfolgt über den Span-8-Pipettierkopf. Ein Self-Refilling-Reservoir (amplius GmbH) ermöglicht die Zufuhr von bis zu vier verschiedenen Lösungsmitteln mit einem Volumen von jeweils 40 ml (3). Über Ultraschallsensoren erfolgt eine permanente Kontrolle des Füllstands; bei Bedarf wird ein automatisches Auffüllen des Reservoirs aktiviert. Dadurch kann der Platzbedarf für die Bereitstellung von Lösungsmitteln und Reagenzien auf dem Deck des i7 minimiert werden. Eine peltiergekühlte Station wurde für die Kühlung der eingesetzten Standards ebenfalls integriert. Die Entfernung kleiner Flüssigkeitsmengen kann über ein 3D Tilting ALP (*automated labware positioner*, amplius GmbH) erfolgen, das eine dreidimensionale Bewegung mit einem Winkel von 15° ermöglicht (4).

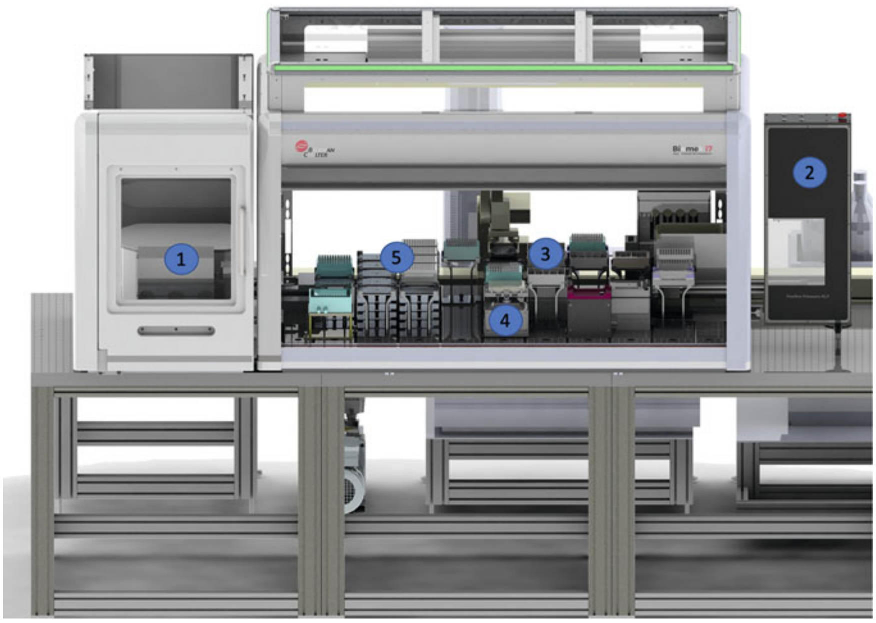

▲ **Abb. 2:** Liquidhandler Biomek i7 für Flüssigkdosier- und Transportprozesse (1), Positive Pressure Extractor für automatisierte Festphasenextraktion (2), Self-Refilling-Reservoir für die Lösungsmittel-/Reagenzienzufuhr (3), 3D-Tilting-ALP für Mischprozesse (4), Deckpositionen für Proben und Labware (5).

Die maximale Anzahl der auf dem Deck zu bearbeitenden Proben ist abhängig von der jeweils genutzten Methode und liegt bei 96 Proben für die Methoden 1 und 2 sowie 144 Proben bei Methode 3, da in diesem Fall keine zusätzlichen SPE-Kartuschen benötigt werden. **Abbildung 2** zeigt das eingesetzte System; die Deckkonfiguration variiert in Abhängigkeit der verwendeten Methode.

Die Proben werden dem Automationssystem in klassischen Safe Lock Vials (Eppendorf) auf einem speziellen Adapter zur Verfügung gestellt (celisca). Für die in Methode 1 durchgeführte Proteinfällung sowie als Probengefäße für die finalen Lösungen sind Standard 2-ml-Vials (Agilent Technologies) erforderlich. Die Standards werden ebenfalls in 2-ml-Vials in einem speziellen Aluminiumrack (celisca) bereitgestellt.

Eine weitere Automatisierung ist möglich, wenn der Liquidhandler mit einem Roboter gekoppelt wird. Damit werden die Entnahme fertig prozessierter Proben sowie die Überführung in das analytische Messsystem möglich. Ferner gelingt über diese Lösung das automatisierte Ein- und Ausschleusen von Labware und Proben.

## Ergebnisse

Die Testung der Automationsmethoden erfolgte mit realen Schweine-Serumproben (FBN, LALLF). Dabei wurden die Gehalte an Vitamin D2 (25(OH)D2) sowie Vitamin D3 (25(OH)D3) bestimmt. **Tabelle 1** stellt die wichtigsten Parameter der drei Methoden gegenüber. Die höchsten Wiederfindungsraten wurden für Methode 3 mit Verwendung der Phree-Platten bestimmt. Die dabei erfolgte Abtrennung der Phospholipide wirkte sich auch positiv auf das verwendete analytische Messsystem aus. Es wurden signifikant

**Tab. 1:** Analysenergebnisse für die automatisierten Methoden.

| | Methode 1 | Methode 2 | Methode 3 |
|---|---|---|---|
| Proteinabtrennung | Zentrifuge | Impact-Platten | Phree-Platten |
| Probenaufreinigung | SPE | SPE | – |
| Max. Probenzahl | 96 | 96 | 144 |
| Wiederfindung [%] | | | |
| Vitamin D2 | 83,31 | 77,76 | 97,36 |
| Vitamin D3 | 82,85 | 79,57 | 94,61 |
| Präzision [%] | | | |
| Vitamin D2 | 3,07–4,71 | 5,31–12,19 | 2,29–8,36 |
| Vitamin D3 | 1,63–3,06 | 2,35–8,23 | 1,69–5,51 |
| Nachweisgrenze [ng/mL] | | | |
| Vitamin D2 | 2,16 | 8,99 | 46,89 |
| Vitamin D3 | 5,81 | 14,95 | 78,21 |
| Bestimmungsgrenze [ng/mL] | | | |
| Vitamin D2 | 4,62 | 20,68 | 98,01 |
| Vitamin D3 | 14,4 | 37,66 | 203,94 |

weniger Kontaminationen der verwendeten Trennsäulen sowie des Massenspektrometers (geringeres Untergrundrauschen) beobachtet. Etwas höhere Standardabweichungen werden für die Methode 2 (Impact-Platten) erhalten, was vermutlich auf eine etwas geringere Effizienz der Proteinfällung zurückzuführen ist. Die Originalmethode mit Zentrifugation weist die besten Werte für die Nachweis- und Bestimmungsgrenzen auf. Methode 3 liegt hier über den Zielwerten, was auf unterschiedliche Elutionsvolumina und damit der Konzentrationen in den finalen Lösungen im Vergleich zu den SPE-basierten Methoden 1 und 2 zurückzuführen ist. Eine weitere Verbesserung der Nachweis- und Bestimmungsgrenzen gelingt hier durch den Einsatz von MS/MS-Verfahren. Der Verzicht auf eine zusätzliche Probenaufreinigung mittels SPE führt zu einer Effektivierung und höheren Durchsätzen.


Danksagung

Die Forschungsarbeiten wurden unterstützt von Beckman Coulter, Indianapolis (USA). Die Autoren danken Dr. Thomas Roddelkopf, DI Lars Woinar, Heiko Engelhardt und Sybille Horn für die Unterstützung bei der Systemintegration sowie der Durchführung der Untersuchungen.

Funding note

Open Access funding enabled and organized by Projekt DEAL.

Kerstin Thurow, Heidi Fleischer und Anna Bach (v. l. n. r.)


Literatur

[1] El-Khoury JM Reineks EZ Wang S Progress of liquid chromatography-mass spectrometry in measurement of vitamin D metabolites and analogues Clin Biochem 2011 44 66 76 10.1016/j.clinbiochem.2010.05.007 20493831
[2] Christakos S Ajibade DV Dhawan P Vitamin D: Metabolism Endocrinol Metab Clin North Am 2010 39 243 253 10.1016/j.ecl.2010.02.002 20511049 PMC2879391
[3] van Schoor NM Lips P Worldwide vitamin D status Best Pract Res Clin Endocrinol Metab 2011 25 671 680 10.1016/j.beem.2011.06.007 21872807
[4] Spedding S Vitamin D and human health 2015 Basel MDPI
[5] Burgess RR Deutscher MP Guide to protein purification 2009 Cambridge, Massachusetts, USA Academic Press
[6] Bruce SJ Rochat B Béguin A Analysis and quantification of vitamin D metabolites in serum by ultra-performance liquid chromatography coupled to tandem mass spectrometry and high-resolution mass spectrometry — a method comparison and validation Rapid Commun Mass Spectrom 2013 27 200 206 10.1002/rcm.6439 23239334
[7] Ding S Schoenmakers I Jones K Quantitative determination of vitamin D metabolites in plasma using UHPLC-MS/MS Anal Bioanal Chem 2010 398 779 789 10.1007/s00216-010-3993-0 20628873 PMC2939348
[8] Duan X Weinstock-Guttman B Wang H Ultrasensitive quantification of serum vitamin D metabolites using selective solid-phase extraction coupled to microflow liquid chromatography and isotope-dilution mass spectrometry Anal Chem 2010 82 2488 2497 10.1021/ac902869y 20151683
